# Enamel Matrix Derivatives as an Adjunct to Alveolar Ridge Preservation—A Systematic Review

**DOI:** 10.3390/dj11040100

**Published:** 2023-04-10

**Authors:** Omid Fakheran, Kai R. Fischer, Patrick R. Schmidlin

**Affiliations:** 1 Department of Periodontics, Dental Implants Research Center, Dental Research Institute, School of Dentistry, Isfahan University of Medical Sciences, 81658 Isfahan, Iran; 2 Department of Oral Surgery and Orthodontics, University Clinic of Dental Medicine and Oral Health, Medical University of Graz, Graz 8010, Austria; 3 Clinic of Conservative and Preventive Dentistry, Division of Periodontology & Peri-Implant Diseases, Center of Dental Medicine, University of Zurich, Plattenstrasse, 11 8032 Zurich, Switzerland

**Keywords:** alveolar ridge preservation, enamel matrix derivatives, bone regeneration, tooth extraction, dental implant

## Abstract

Purpose: To systematically assess the current evidence regarding the adjunctive application of enamel matrix derivatives (EMDs) during alveolar ridge preservation (ARP) following tooth extraction. Methods: A comprehensive literature search was conducted in MEDLINE, Cochrane Library, PsycINFO, Web of Science, Google Scholar, and Scopus to identify relevant randomized controlled clinical trials (RCTs). The primary outcome parameters of this systematic review were histomorphometric and radiographic data; secondary outcomes were the feasibility of implant placement after ARP as well as patient-related outcomes such as postoperative discomfort. Results: The search identified 436 eligible articles published from 2011 to 2022, but only five were ultimately included for data extraction (146 patients). Given the substantial heterogeneity among the included studies, no meta-analysis could be performed. The authors’ qualitative analysis showed marginally improved outcomes regarding an increased percentage of new bone formation after tooth extraction and a reduction in postoperative discomfort. Conclusions: Given the potential value of EMDs in other fields of regenerative dentistry, more consideration should be given to EMDs as an adjunctive treatment option in ARP. However, more well-controlled randomized clinical trials are necessary to evaluate the exact potential and impacts of EMDs.

## 1. Background

Actual decision making in tooth extraction is, in most cases, inevitably related to tooth replacement. In this regard, many factors, such as the available treatment options, advantages and disadvantages of various interventions and, eventually, preferences of the clinician and the patient, are involved [1]. Dental implant therapy is a valuable treatment option to replace teeth and is considered an optimal intervention to restore function and esthetics [2]. It is important to emphasize that this treatment modality requires a comprehensive consideration of biological processes such as remodeling of tissues in extraction sockets and osseointegration of dental implants after surgery, as well as various tissue engineering methods to predictably achieve long-term success [3,4].

The alveolar bone proper and periodontal ligament are tooth-dependent structures. These structures are lost after extraction of teeth, and the volume of the alveolar bone is also significantly diminished because of physiological tissue remodeling and lack of natural stimulation [5]. This situation may lead to substantial loss in bone volume, especially in the anterior dentition, and to difficulties in implant surgery, with the need for bone augmentation, the inability to perform prosthetically driven implant placement, poor aesthetic outcomes, and other clinical and patient-centered complications [6,7]. Accordingly, in a recent systematic review, twenty articles were included in a meta-analysis to estimate the post-extraction dimensional changes in the alveolar ridge after unassisted socket healing [8]. The results revealed that the mean horizontal, vertical mid-facial, and mid-lingual ridge reduction in molar sites was 3.61 mm (95% CI: 3.24–3.98), 1.46 mm (95% CI: 0.73–2.20), and 1.20 mm (95% CI: 0.56–1.83), respectively. This study also concluded that in non-molar sites the mean horizontal, vertical mid-facial, and mid-lingual ridge reduction was 2.54 mm (95% CI: 1.97–3.11), 1.65 mm (95% CI: 0.42–2.88), and 0.87 mm (95% CI: 0.36–1.38), respectively [8]. Alveolar ridge preservation (ARP) is a preventive treatment approach to avoid or at least minimize physiologic bone resorption and to maintain esthetics following tooth extraction [9]. Regarding this treatment modality, various types of graft materials, such as autologous bone chips, alloplasts, xenografts, allografts, autologous blood derivatives, and biologics, have been used. Although xenograft materials and collagen membranes are widely utilized for ARP, substantial controversy in the literature remains with respect to the actual effectiveness of these materials [10,11,12].

To minimize bone dimensional changes and improve the quality and quantity of new tissue in the extraction socket, various growth factors, such as enamel matrix derivatives (EMDs), platelet-derived growth factor, and bone morphogenetic proteins (BMPs), have been suggested as biological adjuncts in ARP modalities in recent years [13,14]. Such biological elements are signaling factors that regulate cell development and growth, and they modulate extracellular matrix formation and also cell proliferation and migration [15]. The incorporation of growth factors in ARP modalities may therefore conceptually provide the opportunity to accelerate new bone formation and, consequently, ridge preservation [16]. A high quality systematic review and meta-analysis compared the overall effect of nine different ARP treatment modalities with tooth extraction alone. The results showed that ARP prevented horizontal bone width (1.99 mm [95% CI: 1.54–2.44; *p* < 0.00001]), mid-lingual bone height (1.99 mm [95% CI: 0.81–1.52; *p* < 0.00001]), and mid-buccal bone height (1.72 mm [95% CI: 0.96–2.48; *p* < 0.00001]) compared to tooth extraction alone [9].

Enamel matrix derivatives (EMDs) contain growth factors that are extracted from tooth buds of piglets and suspended in a polyglycol gel [17]. EMDs contain over 95% amelogenin, with the remainder consisting of enamelin and other proteins [18]. Several studies have shown that EMDs promote periodontal regeneration and new cementum attachments, and amplify antimicrobial actions on various periodontal pathogens [19,20,21].

A novel liquid carrier system for EMDs with improved physico-chemical properties has been recently introduced specifically for bone graft mixing (EMD-liquid, Osteogain^®^, Straumann). The results of an in vivo study based on histomorphometric assessments showed that using Osteogain induced superior mineralized augmentation tissues in standardized pure bone defects in a rabbit GBR model when compared to control (empty) [22].

EMDs induce a regenerative process in the treated tissues through stimulation of local growth factor secretion and cytokine expression. In vitro microarrays studies using primary human bone cells have demonstrated that EMDs contains both TGF-beta and BMP-like growth factors that contribute to the induction of bio-mineralization. EMDs stimulate bone sialoprotein (BSP) gene transcription in osteoblasts by inducing expression of nuclear proteins that bind to the fibroblast growth factor (FGF)-2 response element and TGF-β1 activation element in the BSP gene promoter [23,24].

To date, no ARP approach has been proven to completely eliminate contour changes after tooth extraction, and no clear gold standard exists in relation to volumetric, histological, and implant-related outcomes. As EMDs are effective in improving clinical and histological parameters in periodontal regenerative and plastic surgery procedures, they might also be a valuable adjunct to ARP. Referring to our comprehensive search, there is no systematic review paper in the literature that specifically focused on the clinical and patient-reported outcomes regarding the use of EMDs in ARP.

In vitro studies showed that the combination of EMDs with a bovine-derived natural bone mineral can significantly enhance osteoblast cell adhesion, proliferation, and differentiation [25]. Accordingly, we hypothesized that EMDs mixed with xenografts in ARP may prevent or reduce the dimensional changes of the alveolar ridge and achieve the desired functional and aesthetic outcome for implant-supported restorations. Therefore, the objective of the present systematic review was to investigate the effect of using EMDs as an adjunctive biomaterial in ARP.

## 2. Material & Methods

### 2.1. Search Strategy

**Figure 1:**
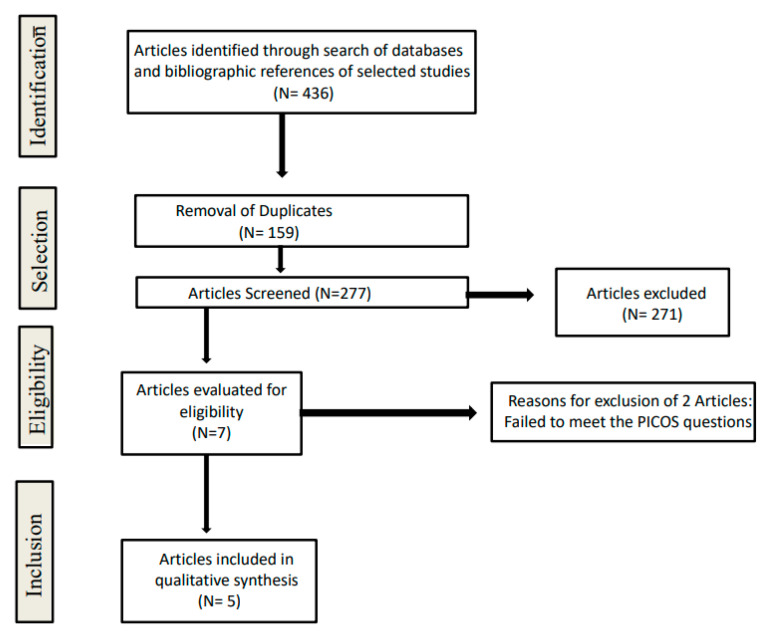
PRISMA flow chart of selection process.

We conducted this systematic review project based on the instructions of the Cochrane Handbook and the final report is written according to the Preferred Reporting Items for Systematic Reviews and Meta-analyses (PRISMA) statement items (Figure 1) [26,27]. We also submitted the protocol of this study to the International Prospective Register of Systematic Reviews (PROSPERO) database (ID: CRD42021269891) (www.crd.york.ac.uk/PROSPERO, (accessed on 27 February 2021)) before starting the project.

We designed the protocol of our systematic search to answer the following focused question: “Following tooth/root extraction in humans, what is the adjunctive effect of using EMDs compared to using xenografts in ARP treatment approaches?” In this regard, the following PICOS strategy was developed: Participants (P) included healthy adult individuals need for tooth extraction before dental implant treatment.

The intervention (I) was the use of an EMD in combination with a xenograft for ARP.

The comparison (C) was to natural healing or ARP with a xenograft.

Outcomes (O) were histologic, histomorphometric, and radiographic results, postoperative discomfort and feasibility of implant placement.

The study (S) was designed for humans, and only randomized controlled clinical trials (RCTs) were evaluated.

### 2.2. Eligibility Criteria

The inclusion criteria were determined as only RCTs of either a parallel design or split-mouth and the use of EMDs in ARP as the test group. The EMD should be used in combination with a xenograft. Only studies that assessed the adjunctive effect of EMDs on ARP were included. There was no limitation on the follow-up duration or the number of patients treated. Any other types of research design, such as animal research or studies reporting on immediate implant placement and papers written in any language other than English, were excluded.

### 2.3. Data Sources & Search Strategy

An electronic literature search was performed using a wide range of computerized databases, including MEDLINE, Cochrane Library, PsycINFO, Web of Science, Google Scholar, and Scopus. These sources were systematically searched between 10 March 2021 and 11 April 2021, with no restrictions on language or publication date.

The following search terms and protocols were used in this systematic review:

((socket [All Fields]) OR (ridge [All Fields])) AND (preservation) [All Fields] AND (enamel matrix derivative [All Fields] OR (Emdogain [All Fields]) OR (amelogenin [All Fields]) OR (dental enamel proteins [All Fields] OR (EMD [All Fields]).

The keywords and search terms were adapted for each database where necessary [28]. We also carried out a comprehensive manual search to cover the references of the included papers and previous review articles. Furthermore, websites that list ongoing clinical trials were also searched (http://clinicaltrials.gov (accessed on 15 March 2021), http://www.centerwatch.com/ (accessed on 20 March 2021) and http://www.clinicalconnection.com (accessed on 22 March 2021)).

### 2.4. Study Selection & Data Extraction

The first stage of selecting the articles was accomplished by two reviewers (O.F. and P.S.) who independently screened (1) titles and (2) abstracts. In the next stage, the same reviewers downloaded and evaluated the full text of all qualified studies. Disagreements were resolved through discussion. Papers that did not meet the eligibility requirements were excluded, and the rationales for exclusion were recorded.

The data were extracted and assimilated on a piloted, standardized data collection sheet. The data were extracted in relation to year of publication, country, measurement methods, patient/tooth characteristics, confounding factors, defect characteristics, surgical procedure, follow-up details, and outcomes related to the aims of this study.

### 2.5. Risk of Bias Assessment

Two reviewers (O.F. and P.S.) independently conducted a risk of bias assessment using “The Cochrane Collaboration’s tool for assessing risk of bias” [29]. The following six domains were assessed: random sequence generation, allocation concealment, blinding of participants and personnel, blinding of outcome assessors, incomplete outcome data, and selective outcome reporting. Each domain was rated as having a high risk of bias, low risk of bias, or unclear according to the Cochrane Handbook recommendations [30]. Any discrepancy between reviewers in quality ratings was resolved by discussion and consensus.

## 3. Results

The initial database search yielded 435 entries, and one article was found by a manual search. No unpublished or ongoing trials were included. After exclusion of duplicates, 277 items were included in the title and abstract screening. Afterwards, seven articles remained to be appraised for eligibility based on the inclusion criteria. Two papers were excluded from the full-text assessment because they did not correspond to the PICO question about intervention groups [31,32]. The final selection consisted of five articles (Figure 1) [33,34,35,36,37]. In view of the marked heterogeneity, no meta-analysis could be conducted; instead, a descriptive data synthesis was performed.

### 3.1. Risk of Bias Assessment

Three studies were classified as having a low risk of bias [34,35,36]. Two of the included studies were classified as having a high risk of bias due to the lack of any reporting regarding the blinding of participants and investigators and blinding of outcome assessment [33,37]. Adequate sequence generation and allocation concealment were reported in all of the included articles. Blinding of participants was mentioned in two of the included studies [34,35] and blinding of outcome assessors in only one study [36]. Furthermore, the completeness of outcome data and absence of selective outcome reporting were considered adequate in all studies. Detailed information is presented in Table S1.

### 3.2. Study Characteristics

The main characteristics and outcomes of the included studies are summarized in Table 1 and Table 2. All the included studies were RCTs and used a parallel design. These studies were conducted in the Republic of Korea, United States of America, Australia, and Argentina. No cohort studies were identified.

Confounding factors such as systemic disease, medication, periodontitis, and smoking were rarely reported. The extraction site distribution among the included studies was heterogeneous. In two investigations, ARP was conducted only in maxillary anterior sockets [34,36]. In another study, first and second molar sockets were assessed [35]. One study did not include any specific tooth area [33]. The latter study included single anterior extraction sockets [37]. The defect morphology around the investigated sockets at the time of extraction varied from 50% buccal bone loss to ≤1 mm buccal bone dehiscence (Table 1).

### 3.3. Intervention Characteristics

All included studies in this review used a combination of an EMD with deproteinized bovine bone mineral with 10% collagen (DBBMC, Geistlich Bio-Oss^®^, Wolhusen, Switzerland) as a carrier and filler material. In two studies, both the test and control groups received coverage with two layers of a native bilayer non-cross-linked resorbable collagen membrane [34,35]; in the remaining reports, the sockets were left uncovered [33,36,37].

In two studies, no flap or incision was used in the surgical procedure [34,35]. However, one of the studies reported full-thickness flaps with vertical incisions as the method of surgery [33]; intrasulcular incisions without any flap elevation were used in the other study [36]. In the most recent study a lingual or palatal flap was elevated and displaced to achieve primary closure without elevating a vestibular flap [37].

In three investigations, amoxicillin 1.5 g/day for five days and a 0.12% chlorhexidine (CHX) mouth rinse for two weeks were administered to the patients [33,34,35]. In another study, a 0.12% CHX mouth rinse for one week and 0.12% CHX gel for the 2nd and 3rd weeks postoperatively were prescribed [36].

The follow-up period was three months in three studies, four months in one study, and six months in the most recent study (Table 1).

### 3.4. Radiographic Results

Three studies measured horizontal and vertical bone height by means of cone beam-computed tomography (CBCT) imaging [34,35,36]. These assessments were conducted at baseline and at four or five months after tooth extraction. The results of all three studies showed no significant differences between the test and control groups regarding horizontal bone width or vertical bone height changes.

### 3.5. Histologic and Histomorphometric Results

Three studies conducted histologic and histomorphometric analyses [33,36,37], with one reporting no significant differences between the control and test groups regarding the percentage of new bone and type of tissues [33]. Based on the results of two other studies, three area fractions (percentage components) were identified in each core sample using a magnification of up to 40x to achieve accurate delineation of each of the components [36,37]. The authors of these studies classified these percentage components as new bone (NB), residual graft (RG), and soft tissue and marrow spaces (STMs). One of these studies reported that in the test group (DBBMC + EMD), 45.1 ± 8.8% of new bone filled the socket, which was significantly higher than the 16.5 ± 6.9% (*p* < 0.00001) of new bone found in the control group (DBBMC only) [36]. In the control group, the socket was occupied by 36.8 ± 8.8% RG, which was significantly higher than the 20.3 ± 7.2% RG found in the test group (*p* < 0.00001). There was also a significantly higher %STM in the control group (%STM control = 46.5 ± 10.4, %STM test = 34.6 ± 13.8, *p* < 0.003).

### 3.6. Postoperative Discomfort

Only two studies reported postoperative discomfort after extraction and ARP [34,35]. One of these clinical trials reported no significant differences among test and control groups regarding the severity of pain, severity of swelling, duration of pain, and duration of swelling [35]. In another paper, however, it was mentioned that the duration of pain and swelling were significantly reduced in the test group (DBBMC + EMD), even though the severity of pain and swelling did not differ between the groups [34].

### 3.7. Feasibility of Implant Placement

Three of five included studies reported the feasibility of implant placement following ARP [33,35,36]. These studies indicated successful placement of implants without any significant differences between the test and control groups (Table 2).

## 4. Discussion

In recent years, several surgical methods have been proposed to preserve alveolar ridge dimensions subsequent to tooth extraction [9,38,39]. In this systematic review we tried to collect and evaluate the available evidence on application of EMD in ARP.

The sockets are usually self-contained following dental extraction. Therefore, the prospective benefits of additional agents, in addition to those for saving space (conventional bone grafts) or preventing soft tissue invasion (commercially available collagen membranes), may be insignificant [38]. This does not inevitably mean that they are useless. Rather, it shows a lack of standardization in their application method [39]. According to this concept, many trials have shown that adjunctive use of various bioactive materials, such as platelet-rich fibrin (PRF) and recombinant human bone morphogenetic protein-2 (rhBMP-2), in the ARP procedure might be beneficial [14,39,40,41,42,43]. In a clinical trial study, the investigators assessed the regenerative ability of rhBMP-2 in tooth extraction sockets with more than 50% buccal dehiscence compared to a placebo collagen carrier [40]. In this study CBCT analysis showed that rhBMP-2 could regenerate a part of buccal bone plate. Moreover, five months later the suitable sites for placement of implants were provided in the test group. The test group showed statistically significant improvements (*p* < 0.05) regarding clinical ridge width (6.0 versus 4.62 mm), radiographic ridge width (6.17 versus 4.48 mm) and clinical buccal plate regeneration (4.75 versus 1.85 mm).

In another split-mouth, randomized, controlled clinical trial the researchers evaluated bone structure and dimensional changes in tooth extraction sites when leucocyte and platelet-rich fibrin (L-PRF) or advanced platelet-rich fibrin+ (A-PRF +) was used in comparison to natural socket healing. [41]. The results of this study revealed that the dimensional changes (palatal and buccal side) at extraction sockets were not significantly different at 1 mm below the crest of both groups (*p* > 0.05). However, for the socket fill, L-PRF (85.2%) and A-PRF+ (83.8%) showed superior values compared to the control (67.9%). Notably, the radiological analysis and histologic evaluations confirmed the existence of more newly formed bone in the PRF groups compared to control sites. However, L-PRF and A-PRF+ sites were not significantly different in this regard. [41].

Nevertheless, all the systematic reviews on using bioactive materials in ARP point to lack of high quality evidence in the literature. Hence, we need more longitudinal studies and RCTs, with low risk of bias, to better understand the advantages and disadvantages of using PRF and rhBMP-2 in alveolar ridge preservation. [42]. Overall, with regard to diversity, comparisons among various adjunctive materials and EMDs in the ARP procedure are complicated.

To date, there have been only a few qualified RCTs evaluating the effect of the adjunctive use of EMDs on ARP, and these studies showed substantial heterogeneity regarding the design and region of extraction sockets. The last published paper related to this topic was the consensus statement on the use of biologics in clinical practice published by the American Academy of Periodontology [42,43]. In this consensus statement, the authors only evaluated two studies regarding the application of EMD in ARP [34,36]. However, in our systematic review, we not only included these two articles, but also included three more studies. Furthermore, this systematic review is the first study focused exclusively on the efficacy of EMD application in ARP.

Thus, it was our intention to summarize and evaluate the available evidence regarding the effect of adjunctive use of EMDs in ARP. Previous studies have indicated the osteogenic potential of EMDs in in vitro and in vivo investigations [16,44,45]. The results of Invivo studies revealed that EMDs can upregulate the expression of osteogenic gene in progenitor cells. [46,47,48,49]. On the other hand, EMDs inhibit bone resorption by affecting osteoclast activities through increased osteoprotegerin (OPG) release and decreased receptor activator of nuclear factor kappa B ligand (RANKL) release [50,51,52]. Furthermore, EMDs appear to stimulate bone cell proliferation and differentiation [18,53]. In general, EMDs enhance the osteogenic capacity of bone marrow and increase mineralized nodule formation. [54].

In three of our included studies, tissue composition was evaluated histomorphometrically via core samples during implant placement [33,36,37]. In one investigation, the authors reported no significant differences regarding the amount of new bone formation [33]. However, this paper did not report any detailed information regarding the socket locations and defect characteristics. It should be noted that only four teeth were included per group in this study. Given the small number of teeth, the statistical assessment of this investigation may not be generalizable [33]. In other two studies, a larger number of teeth were included (21 in each group), and histomorphometric analysis revealed statistically significant differences in terms of the percentage of new bone, residual graft and soft tissue, and marrow spaces between test and control sites [36,37]. The increased amount of new bone in the test group demonstrated that the addition of an EMD increased osteogenic potential. These results are in accordance with the current evidence. Moreover, EMDs can increase expression of vascular endothelial growth factor (VEGF), which is a known angiogenic growth agent. VEGF increases angiogenesis and blood supply and may contribute to bone regeneration within the extraction socket. [46,55,56].

In two of the included investigations, which were conducted by the same research team, the authors reported some patient reported outcomes (PROs) [34,35]. In one of the trials, which included only maxillary incisor sockets, the duration of pain and swelling were significantly reduced in the test group [34]. In the second study focusing on maxillary molars, there were no significant differences in postoperative discomfort criteria between the test and control groups [35]. These results show that EMDs may be beneficial in reducing postoperative discomfort in some cases. However, it is not possible to make definite conclusions regarding the PROs and postoperative discomfort based on these limited data. More clinical trials are required for providing further evidence regarding the PROs. The underlying mechanisms of action of EMDs in dental extraction sockets and surgical wounds are not fully understood. However, some proposed biological actions of EMDs, such as promoting angiogenesis, increasing production of TGF-β1, and stimulating microvascular endothelial cell proliferation and chemotaxis, are considered to be closely related to enhanced early wound healing and reduced pain and swelling duration [57,58,59,60,61].

Given the small number of included studies in this systematic review, our results should be implicated with caution. We could not conduct quantitative analysis in our study due to existence of substantial heterogeneity in clinical measures among the included studies.

Nonetheless, the qualitative analyses of radiographic evaluation, histomorphometric assessments, and postoperative discomfort generally revealed some positive finding for the test groups, though they did not always reach the level of statistical significance. It should also be noted that the results are based on only a few reports.

## 5. Conclusions

Given the limitations of this review and based on the qualitative nature of the analysis, the adjunctive use of EMDs may play a positive role in reducing the duration of postoperative discomfort and increasing the percentage of new bone formation. However, these effects have not been sufficiently scientifically delineated, and no difference in ridge dimension changes or radiographic outcomes has been observed. Therefore, larger multicenter clinical trials and standardized measurement methods are necessary to evaluate the promising role of EMDs in alveolar ridge preservation following tooth extraction.

## Figures and Tables

**Table 1:** Main characteristics of the included studies.

First Author Year of Publication Study Design Country	Measurement Methods	Patients (Teeth) Characteristics	Confounding Factors 1. Smoking 2. Periodontitis	Defect Characteristics 1. Socket Location 2. Defect Morphology	Surgical Management 1. Type of Flap 2. Soft Tissue Management 3. Post Operative Antimicrobials	Follow up 1. Healing Period 2. Number of Drop outs 3. Adverse Events	Test and Control Groups
Nevins 2011 [33] Single center RCT USA	Histological analysis (light microscopy and B-SEM) Histomorphometric analysis	15 patients/ 16 teeth Age: 18–70	1. No 2. N/R	1. N/R 2. N/R	1. Full-thickness with vertical incisions 2. N/R 3. Amoxicillin 1.5 g/day for 5 days, 0.12% CHX for 2 weeks	1. 5 months 2. 0 3. Uneventful healing	T1: DBBMC alone T2: DBBMC + rhPDGF-BB T3: DBBMC + EMD T4: Bone ceramic + EMD
Lee 2019 [34] Single center RCT Republic of Korea	Radiographic analysis (CBCT) Clinical assessment Score of discomfort	32 participants/32 teeth 8 men (56.3%) 14 women (43.8%) Mean age: 55.1 Y Age range: 31–71 Y	1. Current smokers (>10 cigarettes/day) considered as exclusion criteria 2. Stable periodontal status (bleeding on probing < 20% and plaque index < 20%)	1. Maxillary central incisors (N = 16) Maxillary lateral incisors (N = 14) 2. ≤50% buccal bone loss	1. No flap/No incision 2. N/R 3. Amoxicillin 1.5 g/day for 5 days, 0.12% CHX mouthwash tid for 2 weeks	1. 5 months 2. 2/32 (6.3%) Test (N = 1) Control (N = 1) 3. Bleeding: (T = 2, C = 2) Persistent swelling: (T = 2, C = 4) Ulceration: (T = 0, C = 1)	T: DBBMC + EMD + 2 layer RCM C: DBBMC + 2 layer RCM
Lee 2020 [35] Single center RCT Republic of Korea	Radiographic analysis (CBCT) Clinical assessment Score of discomfort	36 participants/36 teeth 18 men (64.3%) 10 women (35.7%) Mean age: 52.9 Y Age range: 22–74 Y	1. Current smokers (>10 cigarettes/day) considered as exclusion criteria 2. Stable periodontal status (bleeding on probing < 25% and plaque index < 25%)	1. Maxillary first molars (n = 21) maxillary second molars (n = 7) 2. ≤50% buccal bone loss	1. No flap/No incision 2. N/R 3. Amoxicillin 1.5 g/day for 5 days, 0.12% CHX mouthwash tid for 2 weeks	1. 5 months 2. 8/36 (22.2%) T1 (N = 2) T2 (N = 2) C (N = 4) 3. Spontaneous bleeding (*p*= 0.803): T1, (n = 9) T2 (n = 9) C (n = 9) Persistent swelling (*p* = 0.661): T1 (n = 9) T2 (n = 9) C (n = 9) Ulceration (*p*= 0.538): T1 (n = 9) T2 (n = 9) C (n = 9)	T1: A: DBBMC + EMD + 2 layer RCM 2: DBBMC + 2 layer RCM C: Empty
Mercado 2021 [36] Single center RCT Australia	Radiographic analysis (CBCT) Histological analysis (light microscopy) Histomorphometric analysis	42 participants/42 teeth T: Mean age: 53.6 ± 10.7 (Female = 66 %) C: Mean age: 51.4 ± 11.3 y (Female = 71%)	1. Current smokers considered as exclusion criteria 2. Stable periodontal status (No probing depth >4 mm and bleeding on probing < 20% and plaque index < 20%)	1. Maxillary anterior tooth 2. Buccal dehiscence ≤ 1 mm present at the time of extraction, no palatal defect	1. Intrasulcular incision/(no flap) 2. FGG3. (0.12% CHX mouthwash) tid for 1 week, 0.12% CHX gel 2nd and 3rd weeks postoperatively	1. 4 months 2. 0 3. Uneventful healing	T: DBBMC + EMD C: DBBMC only
Bonta [37] 2022 Single center RCT Argentina	Histological analysis (light microscopy) Histomorphometric analysis	21 participants/21 teeth No other data provided regarding the patient (teeth) characteristics	1. N/R 2. N/R	1. Single anterior extraction sockets 2. N/R	1. Laterally sliding flap 2. N/R 3. N/R	1. 6 months 2. 0 3. N/R	T1: DBBMC only T2: DBBMC + EMD C: Empty

N/R: not reported; T: test group; C: control group; RCT: randomized controlled trial; FGG: free gingival graft; CHX: chlorhexidine; bid: twice a day; (B-SEM): backscatter scanning electron microscopy; rhPDGF-BB: recombinant human platelet-derived growth factor BB; EMD: enamel matrix derivative; DBBMC: deproteinized bovine bone mineral with 10% collagen; RCM: resorbable collagen membrane; CBCT: cone-beam computed tomography.

**Table 2:** Measurement method and outcomes of the included studies.

First Author	Histomorphometric Results	Histologic Results	Radiographic Results	Postoperative Discomfort	Implant 1. Feasibility of Implant Placement 2. Necessity of Simultaneous Augmentation
Nevins 2011 [33]	Percentage of new bone: T1: 28.3 ± 17.2 T2: 39.6 ± 11.3 T3: 23.9 ± 9.3 T4: 21.4 ± 4.2 No statistically significant differences	Residual DBBMC graft particulate surrounded by new and native bone. The results of group C was consistent with that of group A specimens.	N/R	N/R	1. Placement of implants in all C and T sites with good primary stability 2. N/R
Lee 2019 [34]	N/R	N/R	Three CBCT images: at baseline, 3 and 5 months. No significant differences between the test and control groups at 3 and 5 months were found.	The severity of pain and swelling did not differ between the two groups, but the duration of pain and swelling were significantly reduced in the test group.	1. N/R 2. N/R
Lee 2020 [35]	N/R	N/R	Two CBCT images: at baseline and 5 months after ARP. No significant differences between T1 and T2 were found regarding horizontal width or vertical height changes.	There were no significant differences in following parameters among four groups: Severity of pain Severity of swelling Duration of pain Duration of swelling	1. 22 implants placed after 5 months: T1: (N = 9); T2: (N = 8); C: (N = 5) 2. Ten implants placed with additional bone grafting procedures: OSFE/BAOSFE: T1: (N = 3); T2: (N = 3); C: (N = 3) (SFEL): C: (N = 1)
Mercado 2021 [36]	Three area fractions (percentage components) were identified in each sample core: NB: T: 45.1 ± 8.8% C: 16.5 ± 6.9% (*p* < 0.00001) * RG: T: 20.3 ± 7.2 % C: 36.8 ± 8.8% (*p* < 0.00001) * STM: T: 34.6 ± 13.8 C: 46.5 ± 10.4 (*p* < 0.003) *	The three types of tissues filling the socket (NB, RG, and STM) in both groups.	Two CBCT images: at baseline and 4 months after ARP. No statistically significant differences when comparing the mean RW, BH, and PH between T and C. There was a significantly greater percentage reduction in ridge dimensions RW, BH, and PH in the <1 mm BT group when compared to the ≥1 mm BT group.	N/R	1. All patients received the planned dental implants at least 4 months after the extraction 2. N/R
Bonta 2022 [37]	NB: (*p* < 0.05) * T1: 47.30 T2: 32.27 C: 35.62 RG (%): (*p* > 0.05) T1: 11.61 T2: 18.12 STM: (*p* < 0.05) * T1: 57.21 T2: 34.57 C: 64.38	Presence of healthy lamellar bone in all the groups with osteon formation, and evident lack of inflammatory infiltrate in marrow spaces. Residual DBBMC graft particulate surrounded by new and native bone in T1 and T2 groups.	N/R	N/R	1. Placement of implants in all C and T sites 2. N/R

N/R: not reported; T: test group; C: control group; OSFE/BAOSFE: osteotome sinus floor elevation (OSFE) or bone-added OSFE technique; SFEL: sinus floor elevation by the lateral approach; RW: alveolar ridge width; BH: buccal bone height; PH: palatal bone height; NB: new bone; RG: residual graft; STM: soft tissue matrix; BT: buccal bone thickness; (*): statistically significant difference between test and control groups.

## Data Availability

The datasets used and/or analyzed during the current study are available from the corresponding author upon reasonable request.

## Supplementary Materials

The following supporting information can be downloaded at: https://www.mdpi.com/article/10.3390/dj11040100/s1, Table S1: Risk of bias assessment.

## Abbreviations

EMDs	Enamel matrix derivatives
ARP	Alveolar ridge preservation
RCTs	Randomized controlled clinical trials
BMPs	Bone morphogenetic proteins
PRISMA	Preferred Reporting Items for Systematic Reviews and Meta-Analyses
PROSPERO	International Prospective Register of Systematic Reviews
DBBMC	Deproteinized bovine bone mineral with 10% collagen
NB	New bone
RG	Residual graft
STMs	Soft tissue and marrow spaces
CHX	Chlorhexidine
CBCT	Cone beam-computed tomography
rhBMP-2	Recombinant human bone morphogenetic protein-2
PRF	Platelet-rich fibrin
L-PRF	Leucocyte and platelet-rich fibrin
A-PRF+	Advanced platelet-rich fibrin+
