# Supplementary material for: Enamel Matrix Derivatives as an Adjunct to Alveolar Ridge Preservation—A Systematic Review
Source: Dent J (Basel). 2023 Apr 10;11(4):100. doi: 10.3390/dj11040100 (PMC10137019; doi:10.3390/dj11040100)
Supplement: Supplementary file 1 [file dentistry-11-00100-s001.zip › dentistry-2110383-supplementary.pdf]

Table S1: Risk of bias assessment

	Random sequence generation	Allocation concealment	Blinding of participants and personnel	Blinding of outcome assessment	incomplete outcome data	Selective reporting	Other Bias	overall risk of bias
**Nevins 2011**	+	+	-	-	+	+	?	High Risk of Bias
**Lee 2019**	+	+	+	?	+	+	+	Low Risk of Bias
**Lee 2020**	+	+	+	?	+	+	+	Low Risk of Bias
**Mercado 2021 Single center RCT Australia**	+	+	_	+	+	+	+	Low Risk of Bias
**Bonta 2022 Single center RCT Argentina**	+	+	?	?	+	+	+	High Risk of Bias
